# Perfusate Liver Arginase 1 Levels After End-Ischemic Machine Perfusion Are Associated with Early Allograft Dysfunction

**DOI:** 10.3390/biomedicines13010244

**Published:** 2025-01-20

**Authors:** Giuseppina Basta, Serena Babboni, Daniele Pezzati, Serena Del Turco, Emanuele Balzano, Gabriele Catalano, Lara Russo, Giovanni Tincani, Paola Carrai, Stefania Petruccelli, Jessica Bronzoni, Caterina Martinelli, Simona Palladino, Arianna Trizzino, Lorenzo Petagna, Renato Romagnoli, Damiano Patrono, Giandomenico Biancofiore, Adriano Peris, Chiara Lazzeri, Davide Ghinolfi

**Affiliations:** 1 Institute of Clinical Physiology, National Research Council (CNR), Via Moruzzi 1, 56124 Pisa, Italy; 2 Division of Hepatic Surgery and Liver Transplantation, Azienda Ospedaliera Universitaria Pisana, Via Paradisa 2, 56124 Pisa, Italy; 3 General Surgery 2U-Liver Transplant Unit, Azienda Ospedaliero Universitaria Città della Salute e Della Scienza di Torino, University of Torino, Corso Bramante 88-90, 10126 Torino, Italy; 4 Department of Anesthesia and Critical Care Medicine, Azienda Ospedaliero-Universitaria Pisana, 56124 Pisa, Italy; 5 Tuscany Regional Transplant Authority, Centro Regionale Allocazione Organi e Tessuti (CRAOT), 50134 Florence, Italy

**Keywords:** DCD, donations after circulatory death, D-HOPE, dual hypothermic oxygenated machine perfusion, NMP, normothermic regional perfusion, FMN, flavin mononucleotide, EAD, early allograft dysfunction, ARG-1, arginase 1

## Abstract

**Background/Objectives**: The rising use of liver grafts from donation after circulatory death (DCD) has been enabled by advances in normothermic regional perfusion (NRP) and machine perfusion (MP) technologies. We aimed to identify predictive biomarkers in DCD grafts subjected to NRP, followed by randomization to either normothermic machine perfusion (NMP) or dual hypothermic oxygenated perfusion (D-HOPE). **Methods**: Among 57 DCD donors, 32 liver grafts were transplanted, and recipients were monitored for one week post-transplant. Biomarkers linked with oxidative stress, hepatic injury, mitochondrial dysfunction, inflammation, regeneration, and autophagy were measured during NRP, end-ischemic MP, and one week post-transplant. **Results**: Arginase-1 (ARG-1) levels were consistently higher in discarded grafts and in recipients who later developed early allograft dysfunction (EAD). Specifically, ARG-1 levels at the end of MP correlated with markers of hepatic injury. Receiver operating characteristic analysis indicated that ARG-1 at the end of MP had a good predictive accuracy for EAD (AUC = 0.713; *p* = 0.02). Lipid peroxidation (TBARS) elevated at the start of NRP, declined over time, with higher levels in D-HOPE than in NMP, suggesting a more oxidative environment in D-HOPE. Metabolites like flavin mononucleotide (FMN) and NADH exhibited significant disparities between perfusion types, due to differences in perfusate compositions. Inflammatory biomarkers rose during NRP and NMP but normalized post-transplantation. Regenerative markers, including osteopontin and hepatocyte growth factor, increased during NRP and NMP and normalized post-transplant. **Conclusions**: ARG-1 demonstrates strong potential as an early biomarker for assessing liver graft viability during perfusion, supporting timely and effective decision-making in transplantation.

## 1. Introduction

Organ scarcity is a global issue limiting transplant expansion. To address this problem, organs from suboptimal donors, such as donors suffering circulatory death (DCD), are increasingly used, driven by the integration of innovative technologies such as normothermic abdominal regional perfusion (NRP) and ex situ mechanical perfusion (MP), leading to improved transplant outcomes and reduced post-transplant complications [1,2]. MP represents an innovative advance in organ transplantation, particularly in the context of liver viability assessment. By providing a controlled environment to assess organ function before transplantation, this technique has the potential to revolutionize the decision-making process for organ utilization. Although there is an ongoing debate on the optimal ex situ perfusion system [2], MP allows physicians to more accurately determine the suitability of liver grafts from marginal or extended criteria donors, significantly reducing organ rejection rates. In addition, the physiological support offered by MP helps to mitigate ischemic damage, reducing the likelihood of graft failure after transplantation. For this reason, end-ischemic MPs are emerging as indispensable tools for optimizing transplant outcomes and expanding the pool of available organs [2].

The prerequisite for an accurate viability assessment is the availability of reliable biomarkers of liver damage and function, ideally employed interchangeably during any MP technique, including NRP [3,4,5,6,7].

The identification of reliable biomarkers for the assessment of liver injury and function is a significant and ongoing challenge. Current efforts focus on discovering markers that accurately reflect injury, regeneration, and metabolic activity [3,4,5,6,7]. Such advances aim to improve diagnostic accuracy, enabling earlier and more targeted interventions. In the context of liver transplantation (LT) and disease management, these biomarkers have the potential to improve donor organ assessment, optimize recipient outcomes, and facilitate monitoring of therapeutic efficacy.

In DCD grafts, organ damage arising from warm and cold ischemia contributes to ischemia-reperfusion injury (IRI), which is characterized by oxidative stress, depletion of adenosine triphosphate, and mitochondrial dysfunction [8]. Early allograft dysfunction (EAD) is a frequent complication during the first postoperative week after orthotopic LT and is considered a consequence of IRI. The perfusate level of mitochondrial flavin mononucleotide (FMN) has been recently identified as a potential biomarker of graft damage and function during HOPE [9,10,11], whereas other parameters of graft function, including lactate clearance, glucose metabolism, and pH maintenance, represent the backbone of liver viability assessment during NMP [12].

IRI-induced hepatocyte damage leads to the release of various cytosolic enzymes. ARG-1 is an enzyme primarily expressed in the liver, where it plays a crucial role in ammonia detoxification by catalyzing the final step of the urea cycle, converting arginine into ornithine and urea [13]. During liver transplant, reduced ARG-1 levels increase L-arginine availability for NOS, raising NO production. Excessive NO can react with superoxide to form peroxynitrite, a potent oxidant that induces oxidative stress and tissue damage. This process exacerbates IRI and contributes to EAD [14,15].

Additionally, aside from its role in tissue damage caused by depletion during IRI, ARG-1—due to its notably short half-life [16]—may serve as a timely and reliable biomarker for assessing graft function NRP, MP, and post-transplantation. The more rapid decline of this marker may better indicate the liver’s recovery from injury, providing added value compared to the usually employed transaminases [17].

While many studies have focused on indicators of mitochondrial dysfunction, limited knowledge exists regarding the use of additional liver parenchymal enzymes with more early circulation elevation compared to transaminases for assessing liver viability [16,18]. To obtain a more precise assessment of liver damage and recovery of DCD grafts undergoing NRP followed by either NMP or D-HOPE, we concurrently analyzed a range of biomarkers associated with oxidative stress, mitochondrial dysfunction, inflammation, and tissue regeneration.

We highlighted the performance and association of ARG-1 with patient EAD outcomes compared to other biomarkers. We also underlined the limitations of FMN fluorescence detection as a mitochondrial damage biomarker, especially its dependency on perfusate or plasma composition, underscoring its affordability and simplicity but limited universal applicability.

## 2. Materials and Methods

### 2.1. Study Design

This study was part of the DCDNet Study, a pilot, randomized, prospective trial with the primary objective of comparing the impact of D-HOPE versus NMP on the outcome of LT patients receiving DCD liver grafts after normothermic regional perfusion NRP (name of the registry: Comparison of Hypothermic Versus Normothermic Ex-vivo Preservation (DCDNet); trial registration number: Clinicaltrial.gov #NCT04744389; date of registration: 15 December 2020). The main focus of this study was to identify, through an exploratory biomarker analysis, the potential differences in DCD grafts preserved using NRP followed by NMP versus NRP followed by D-HOPE.

All research was conducted in accordance with the principles of the Declarations of Helsinki and Istanbul and was approved by the local ethical committee (Comitato Etico Area Vasta Nord-Ovest of the Azienda Ospedaliero Universitaria Pisana, CEAVNO approval#17848). Donor selection details are reported in the Supplementary Materials.

### 2.2. Normothermic Regional Perfusion, Ex Situ Machine Perfusion, and Outcome

**Figure 1:**
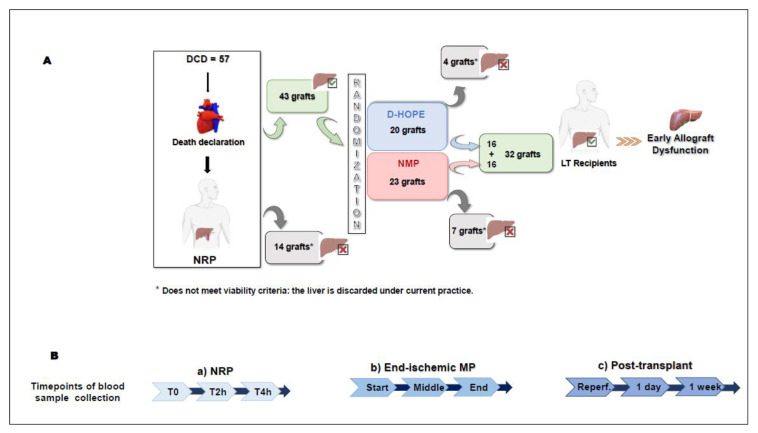
(**A**) A schematic diagram of the donor and patient selection. (**B**) A timeline of blood sample collection: (**a**) at the initiation of NRP (**T0**), 2 h after initiation (**T2h**), and 4 h after initiation (**T4h**); (**b**) at the beginning, midpoint, and conclusion of the end-ischemic MP procedure; (**c**) in recipients: immediately post-reperfusion, one day after liver transplantation, and one week after liver transplantation.

The flowchart of the study is shown in Figure 1A. Details of NRP, MP, and transplantation are specified in the Supplementary Materials, as well as key definitions and outcome measures.

### 2.3. Sample Collection and Analysis

Blood samples were collected at 0, 2, and 4 h of NRP, whereas tissue specimens were collected at the end of NRP. During NRP and end-ischemic MP, a routine biochemical panel including aspartate-aminotransferase (AST), alanine aminotransferase (ALT), glucose, lactate, creatinine, pH, HCO_3_^−^, pO_2_, pCO_2_, and electrolytes (Na, Cl, K, and Ca) was assessed every hour.

Blood samples were collected for study-specific analytes in EDTA at designated times, as illustrated in Figure 1B: (i) at the initiation of NRP (T0), 2 h after initiation (T2h), and 4 h after initiation (T4h); (ii) at the beginning, midpoint, and conclusion of the end-ischemic MP procedure; (iii) in recipients: before reperfusion, immediately post-reperfusion, one day after LT, and one week after LT.

Liver biopsy was performed using a 14G True-Cut needle at the NRP end and stored in Allprotect^®^ Tissue Reagent (Qiagen, S.p.A, Milan, Italy) at −80 °C until use. All sample analyses were performed in a blinded manner

### 2.4. Tissue Quantitative Reverse Transcription Polymerase Chain Reaction (RT-qPCR) of Autophagic Biomarker Expression

Detailed descriptions of this methodology applied on liver tissue specimens collected at the end of NRP are described in Supplementary Materials.

### 2.5. Soluble Biomarker Assays

A panel of soluble biomarkers relevant to inflammation, injury, and regeneration hepatic profiles was evaluated using custom-designed immunoassays based on Luminex xMAP Technology (MILLIPLEX, EMD Millipore Corporation, Billerica, MA, USA) as previously described [19]. Data acquisition was performed using MAGPIX Luminex xMAP technology for Median Fluorescence Intensity and analyzed using Belysa Immunoassay Curve Fitting software version 1.2.0 (Sigma-Aldrich, St. Louis, MO, USA), with a 5-parameter model. The list of the measured analytes, their ranges, and sensitivities are provided in Table S1.

### 2.6. Quantification of Lipid Peroxidation Products, FMN, NADH, Fumarate, and Succinate

The levels of lipid peroxidation products were determined by measuring thiobarbituric acid reactive substances (TBARS). TBARS levels were measured in plasma and perfusate samples using a colorimetric method by measuring the OD values at 530–540 nm, according to the manufacturer’s specifications (E-BC-K298-M, Elabscience Biotechnology Co., Ltd., Wuhan, China). The plasma TBARS values in a population of 20 healthy subjects were 2.5 ± 0.15 µM.

The levels of both FMN and NADH (NADPH) in the plasma and perfusate samples were determined by fluorescence spectroscopy using a microplate fluorescence reader (Tecan Infinite^®^ 200 PRO, Tecan Group Ltd., Männedorf, Switzerland). The FMN fluorescence was read at 450 nm excitation and 550 nm emission, while the fluorescence of NADH was read at 360 nm excitation and 460 nm emission wavelengths. The FMN spectra were calibrated using the FMN standard [19]. The FMN levels of the NMP and D-HOPE perfusion fluids before the start of perfusion were 1410 ± 104 ng/mL and 12 ± 5 ng/mL, respectively.

Fumarate and succinate were analyzed by colorimetric assays (Sigma-Aldrich, MAK060 and MAK184 respectively), according to the manufacturer’s specifications [19].

### 2.7. Statistical Analysis

Continuous variables are reported as mean ± standard deviation (SD) or median (interquartile range), while categorical variables are presented as counts (frequencies). The Kolmogorov–Smirnov test was used to assess normality, and the significance threshold for all analyses was set at *p* < 0.05. Categorical variables were analyzed using a chi-square or Fisher’s exact test, while an independent samples t-test and Mann–Whitney U-test were used for normally and non-normally distributed data, respectively. Pearson’s correlation coefficient was used to explore correlations between the variables. A mixed design (3 × 2) 2-way ANOVA was conducted to identify the main effects of NRP, end ischemic MP, and LT on dependent variables across the three time points, between the groups, and the interaction between group and time. A Friedman’s test with Bonferroni correction was performed within each group across various time points. Due to the small sample size a post hoc power analysis was conducted to determine whether we had a large enough sample size to be able to detect differences between EAD (*n* = 9) and non-EAD groups (*n* = 23). The observed effect size for the difference in ARG-1 levels between these groups at the end of MP resulted in a calculated power >0.80, indicating that the sample size was sufficient to detect a statistically significant difference at the 0.05 significance level.

Receiver operating characteristic (ROC) analysis was performed to evaluate the diagnostic performance of ARG-1 at the end of MPs. The data were analyzed using SPSS 26 (SPSS, Chicago, IL, USA).

## 3. Results

### 3.1. DCD Donors and Transplantation

Among the 57 DCD donors undergoing NRP, 14 liver grafts were discarded during NRP whereas 43 were considered eligible for transplant and were randomized to NMP (n = 23) or D-HOPE (n = 20). Four livers were discarded during D-HOPE, whereas seven were discarded during NMP. After selection, 32 liver grafts were successfully transplanted (Figure 1A).

Baseline donor and recipient characteristics, and clinical outcomes are summarized in Table 1. The dynamic changes in traditional biomarkers including AST, ALT, and lactate levels during NRP, end-ischemic MPs, and until a week post-transplant are shown in Figure S1.

### 3.2. Dynamic Changes and Performance of Early-Releasing Enzymes (ARG-1 and GST-α) Across Machine Perfusion and Post-Transplant

**Figure 2:**
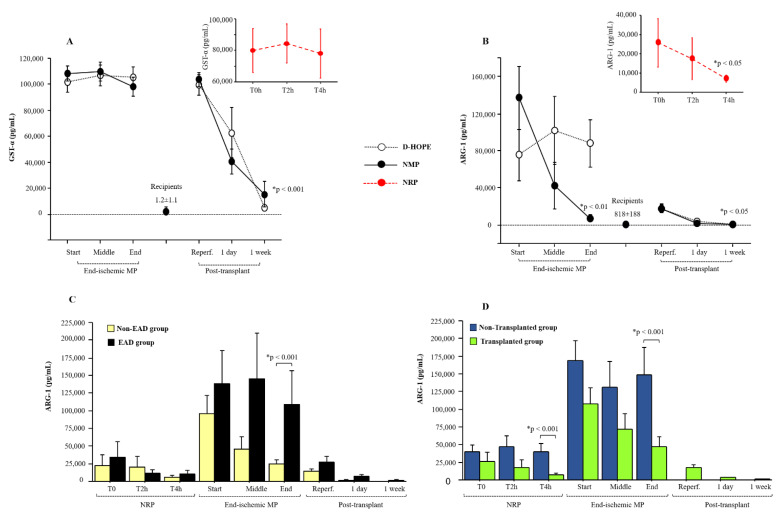
Dynamic change of GST-α (**A**) and ARG-1 (**B**) during NRP, end-ischemic MPs and in recipients until a week post-transplant. Values are presented as mean ± SE. * Significant changes over time within each group (Friedman’s test with Bonferroni correction). Only values of *p* < 0.05, considered as significant, are reported. (**C**) Dynamic change of ARG-1 levels between the EAD group and non-EAD group during NRP, end-ischemic MPs of grafts and in recipients until a week post-transplant. (**D**) Differences in ARG-1 levels between the non-transplanted group and transplanted group during NRP and end-ischemic MPs of grafts.

We measured ARG-1 and GST-α levels, along with all circulating biomarkers analyzed in this study, from the time of organ retrieval up to one week post-transplant, at the intervals specified in Figure 1B. The levels of GST-α remained very high during both NRP and ex situ machine perfusion (Figure 2A). Due to its high half-life, GST-α levels dropped to normal levels only one week after LT (Figure 2A).

Compared to the baseline levels of recipients (818 ± 188 pg/mL), ARG-1 plasma levels were elevated at the start of NRP and dropped at the end of perfusion (Figure 2B). We observed that the levels of ARG-1 decreased in NMP but tended to remain relatively high during D-HOPE, as depicted in Figure 2B. At the end of ex situ MP, ARG-1 perfusate levels correlated with lactate (r = 0.574, *p* < 0.001), ALT (r = 0.427, *p* < 0.05), fumarate (r = 0.704, *p* < 0.001), and succinate (r = 0.781, *p* < 0.001) levels. Elevated plasma ARG-1 levels returned to normal one day after transplantation in the recipients (Figure 2B).

### 3.3. Clinical Outcome

The LT operations were uneventful. No cases of primary nonfunction, vascular complications or ischemic cholangiopathy were reported. There were four cases of biliary leakage after the T-tube insertion that were treated with endoscopic retrograde cholangiography and pancreatography. Twelve patients had postreperfusion syndrome and four had acute kidney injury. There were nine cases of EAD (28.1%). No significant differences in postoperative hospitalization or complications were found based on the type of MP perfusion. Detailed postoperative outcome data are reported in Table 1.

Importantly, ARG-1 levels in the perfusate during and after ex situ graft perfusion were higher in recipients who developed EAD than in those who did not (Figure 2C). In the EAD group, ARG-1 plasma levels persisted higher up to one day after transplant compared to the non-EAD group (Figure 2C). As expected, ARG-1 levels were higher in non-transplanted grafts than in transplanted grafts at the end of both NRP and MP (Figure 2D). Furthermore, discarded grafts at the end of D-HOPE showed higher ARG-1 levels compared to those at the end of NMP (Figure S2).

**Figure 3:**
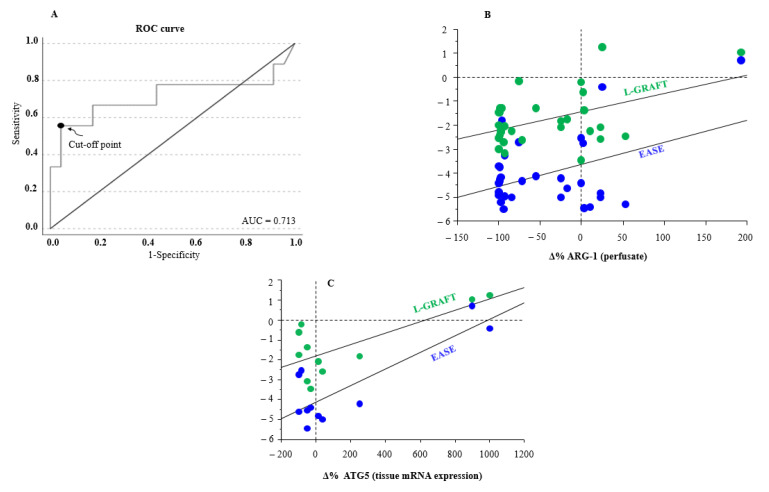
(**A**) ROC curve and sensitivity/specificity graph for ARG-1 concentration differentiating groups at the end of ex situ MP. The threshold for differentiation between EAD group and non-EAD group was 77,471 pg/mL, with a sensitivity of 55.6% and a specificity of 99.9%. AUC, area under the ROC curve; ROC, receiver operator characteristic. (**B**) Correlation between Δ% ARG1 during end-ischemic MPs [(T_end_ − T_start_)/T_start_ × 100] and L-GRAFT (r = 0.481, *p* < 0.01), and EASE (r = 0.448, *p* < 0.05). (**C**) Correlation between Δ% ATG5 tissue mRNA expression during end-ischemic MPs [(T_end_ − T_start_)/T_start_ × 100] and L-GRAFT (r = 0.734, *p* < 0.01), and EASE (r = 0.830, *p* < 0.001) in D-HOPE group.

The diagnostic accuracy of ARG-1 levels at the end of ex situ MP in relation to the diagnosis of EAD was assessed using the receiver operating characteristic (ROC) method. The ROC curve showed an AUC of 0.713 (95% CI: 0.538–1; *p* = 0.02) (Figure 3A). The threshold for differentiation between the EAD group and non-EAD group, assessed using the Youden method, was 77,471 pg/mL, with a sensitivity of 55.6% and specificity of 99.9%.

Furthermore, an association of both EASE and L-GRAFT risk scores with the percentage change of ARG-1 at the end of ex situ MP was observed (Figure 3B).

Correlation analysis highlighted an interesting association between the percentage delta of tissue autophagy related 5 (ATG5), a protein that plays a critical role in the process of autophagy, with both early allograft failure simplified estimation (EASE) and liver graft assessment following transplantation (L-GRAFT) risk scores, only in D-HOPE Figure 3C.

### 3.4. Dynamic Changes of Oxidative Stress Burst

**Figure 4:**
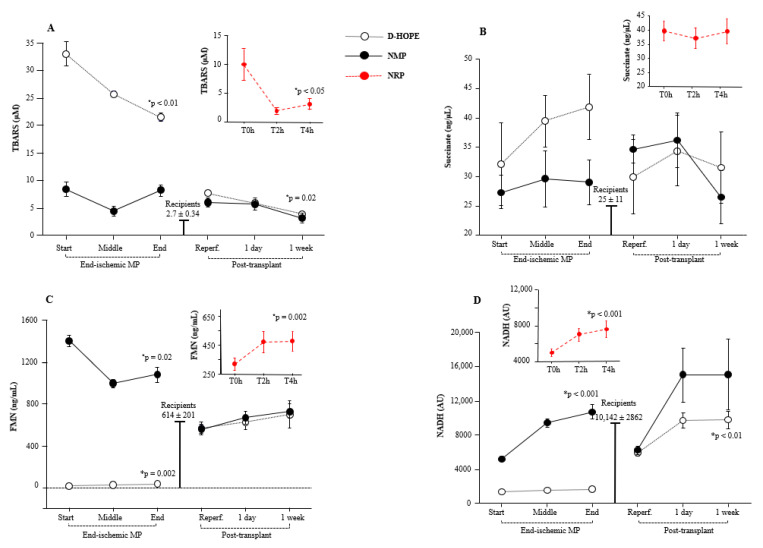
Dynamic change of: (**A**) TBARS, (**B**) succinate, (**C**) FMN, and (**D**) NADH during NRP, end-ischemic MPs and in recipients until a week post-transplant. Values are presented as mean ± SE. * Significant changes over time within each group (Friedman’s test with Bonferroni correction). Only values of *p* < 0.05, considered as significant, are reported.

We quantified the plasma and perfusate lipid peroxidation products, which are indicators of cellular oxidative stress, by measuring TBARS. In comparison to the baseline levels of recipients (levels corresponding to those of healthy subjects), TBARS levels were elevated at the initiation of NRP but declined thereafter (Figure 4A). In contrast, TBARS levels in the perfusate were significantly higher in the D-HOPE group than in the NMP group from the start of perfusion. Although they decreased throughout perfusion, TBARS levels in D-HOPE remained significantly higher than in NMP (Figure 4A). In the recipient, TBARS levels were elevated following post-transplant reperfusion, compared to the recipient baseline values, and normalized only after one week (Figure 4A).

### 3.5. Response of Cellular Energy Metabolism: Dynamic Changes of Succinate, Fumarate, FMN, and NADH

Levels of mitochondrial succinate, whose accumulation represents a key event of hepatic IRI, were elevated in the plasma of donors at the initiation of NRP and remained substantially stable thereafter. During NMP, succinate perfusate levels remained stable (Figure 4B), but progressively increased during D-HOPE. The gap between the two perfusion approaches was marked at the end of perfusion, as shown in Figure 4B. After LT, succinate levels showed an immediate elevation following reperfusion and normalized only after one week (Figure 4B). Compared with the baseline levels of recipients, fumarate plasma levels were elevated at the start of NRP and the start of NMP (Figure S3A). However, at the end of MP perfusion, these values were equal to the D-HOPE values (Figure S3A). They remained unchanged one week after transplantation (Figure S3A).

FMN levels increased during NRP but were significantly lower than the basal levels in recipients (Figure 4C). During ex situ MP, the initial FMN levels were surprisingly over 200 times higher in the NMP perfusate (median 1364 ng/mL) than in D-HOPE (median 6.5 ng/mL) (Figure 4C).

Spectrophotometric analysis of the pre-perfusion solutions of D-HOPE and NMP revealed that the two fluids differed significantly in their FMN content. This marked difference between the two fluids was due to the supplementation of the NMP perfusate with riboflavin B2, a precursor of FMN and FAD. Riboflavin and FMN exhibited identical absorption spectra, which is why riboflavin was used as the standard for the FMN curve. Similarly, donor and recipient plasma FMN values were hundreds of times higher than those in D-HOPE perfusate due to the presence of riboflavin B2 in the plasma (Figure 4C). Based on these considerations, we analyzed the two curves separately and observed a decrease in FMN levels during NMP (*p* = 0.0002) and an increase during D-HOPE (*p* = 0.02) (Figure S3B). Since riboflavin was not initially contained in the D-HOPE perfusion fluid, the absorbance values in D-HOPE reflect the release of FMN, whereas the decrease in absorbance during NMP can be attributed to riboflavin consumption throughout perfusion. However, the high riboflavin B2 concentration in NMP fluids could mask FMN changes. Therefore, despite the decrease in absorbance during NMP, we could not discriminate whether FMN was decreasing or increasing because the impact of riboflavin on the read absorbance was much larger and made the detection of low changes in FMN inaccurate. Post-transplant FMN plasma levels were consistent with the basal levels of both donors and recipients (Figure 4C).

NADH, such as FMN or riboflavin, is also found in the circulation. Accordingly, owing to the different compositions of the fluids, the baseline NADH values were found to be elevated in the NMP group compared to the D-HOPE group and were comparable to the baseline values of donors/recipients (Figure 4D). Owing to the increased metabolism during NMP, the discrepancy between NMP and D-HOPE widened during perfusion (Figure 4D). Post-transplant NADH plasma levels were comparable to baseline levels observed in recipients and showed a tendency to increase one week after LT (Figure 4D).

### 3.6. Dynamic Changes of Inflammation and Regeneration Measurements

**Figure 5:**
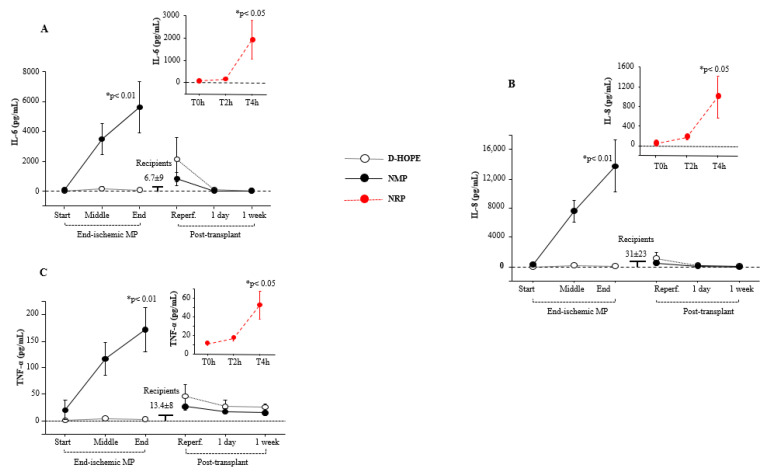
Dynamic change of: (**A**) IL-6, (**B**) IL-8, and (**C**) TNF-α during NRP, end-ischemic MPs and in recipients until a week post-transplant. Values are presented as mean ± SE. * Significant changes over time within each group (Friedman’s test with Bonferroni correction). Only values of *p* < 0.05, considered as significant, are reported.

The levels of the inflammatory interleukin-6 (IL-6), IL-8, and tumor necrosis factor-α (TNF-α) biomarkers increased during NRP and NMP (Figure 5A–C), while they remained unchanged during D-HOPE. They returned to normal levels one day after transplantation (Figure 5A–C).

**Figure 6:**
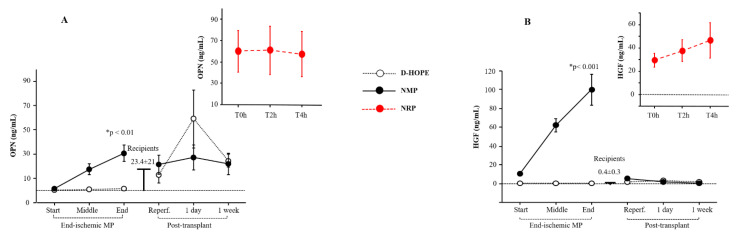
Dynamic change of: (**A**) OPN and (**B**) HGF during NRP, end-ischemic MPs and in recipients until a week post-transplant. * Significant changes over time within each group (Friedman’s test with Bonferroni correction). Only values of *p* <0.05, considered as significant, are reported.

Regeneration biomarkers such as osteopontin (OPN) and hepatocyte growth factor (HGF) were elevated at the start of NRP compared to the recipient baseline values (Figure 6A,B). OPN and HGF levels were enhanced only during NMP (Figure 6A,B), while they remained unaltered during D-HOPE (Figure 6A,B). Post-transplant OPN levels fluctuated and returned to normal values within a week, while HGF levels remained consistently low (Figure 6A,B).

## 4. Discussion

Despite limited clinical trials, MP shows promise in assessing liver viability, reducing discard rates, and avoiding failing grafts [20]. MPs allow the evaluation of organ function, but there are still no reliable biomarkers to monitor liver damage and function in a precise and timely manner. To enhance the precision of liver damage assessment, two innovative liver cytosolic markers were investigated: ARG-1 and GST-α [17,18,19,21]. GST-α has a shorter half-life than transaminases but longer than ARG-1 [18]. These enzymes are particularly suitable in this context thanks to their rapid release into circulation and shorter half-life compared to traditional transaminases, allowing a more timely and precise evaluation of the progress of liver damage.

ARG-1 was found to be more reliable than GST-α, because, in addition to being released early, it also declined more quickly [16]. The relationship between higher ARG-1 levels at the end of ex situ MP and EAD incidence suggests that the monitoring of ARG-1 during the LT procedure could provide significant and clinically useful information together with or independently of the ALT and lactate levels.

The rapid release of ARG-1 allows early detection of cellular damage, while its rapid clearance enables timely recognition when active cellular damage has ceased. Hence, owing to its early release and remarkably brief half-life, ARG-1 has emerged as a hepatic enzyme with a stronger potential for predictive characteristics in transplant outcomes [13,14]. Furthermore, its release can be detrimental because, once in circulation, ARG-1 actively metabolizes circulating L-arginine, inducing its exhaustion. By reducing L-arginine availability, ARG-1 limits the production of nitric oxide and may negatively impact hepatic blood flow [13,22,23]. We demonstrated that ARG-1 levels measured at the end of MPs could predict EAD in LT patients. Another intriguing finding is that ARG-1 levels were higher in non-transplanted grafts discarded after MP than in transplanted, offering valuable insights into the role of ARG-1 as a potential biomarker for graft viability. The elevated ARG-1 in discarded grafts could reflect more advanced or irreversible hepatic injury, suggesting that ARG-1 may serve as an indicator of compromised cellular integrity or significant metabolic dysfunction that renders the graft unsuitable for transplantation.

Additionally, this differential ARG-1 expression implies that ARG-1 could be used prospectively to identify marginal or high-risk grafts before transplantation, supporting clinical decision-making during MP by allowing clinicians to assess the real-time status of liver injury and recovery potential.

The results about TBARS levels indicate that NMP may be more effective than D-HOPE in managing oxidative stress, as shown by significantly lower TBARS levels. Elevated TBARS levels in D-HOPE suggest sustained lipid peroxidation, which could compromise cellular integrity and mitochondrial function. Sustained oxidative stress in D-HOPE could arise from suboptimal oxygenation and reduced mitochondrial activity, leading to incomplete ROS scavenging. This underscores a critical limitation in managing oxidative stress effectively. Inadequate oxygen delivery and impaired mitochondrial function can exacerbate oxidative damage, compromising cellular integrity and organ viability. NMP addresses these challenges by providing physiological temperature conditions that restore robust metabolic activity. This approach enhances mitochondrial function, optimizes oxygen utilization, and facilitates the delivery of critical antioxidants. By mitigating oxidative stress more effectively, NMP offers a superior platform for organ preservation and repair, potentially enhancing graft viability and recipient outcomes.

Schlegel et al. [6] demonstrated that rat livers experiencing irreversible graft injury, primary nonfunction, or cholangiopathy initially exhibited elevated levels of FMN and NADH in the perfusate during HOPE. A recent international study [11] confirmed the predictive value of FMN released during HOPE, supporting better risk stratification of injured livers before implantation. Moreover, in an experimental study, the superiority of D-HOPE over NMP was underlined, mainly relying on monitoring FMN values in the perfusate [24]. However, as clarified in our study, FMN, derived from riboflavin (B2) naturally present in blood, is also added to NMP perfusion solutions as an additive. Consequently, when assessing the predictive value of FMN during D-HOPE or NMP, it is crucial to acknowledge that the perfusate compositions in D-HOPE and NMP are different. Only in D-HOPE, the evaluation of mitochondrial damage through perfusate FMN monitoring is free of confounding factors and may predict clinical outcomes after transplantation.

The increase of perfusate NADH levels during NMP, compared to D-HOPE, can likely be attributed to the reactivation of metabolic and inflammatory processes, immunological responses, and alterations in cell permeability, which may collectively contribute to the extracellular release of NADH [25,26]. In brief, while assessing the release of FMN and NADH provides a fast and cost-effective method for evaluating mitochondrial metabolism and cumulative graft damage during donor liver D-HOPE, prudent and cautious application of these markers during NMP is recommended.

Careful analysis of studies reporting liver-damaging effects of autophagy reveals that excessive autophagy, like an inflammatory response, may harm cells by mistakenly targeting normal organelles or proteins [27,28,29]. Our findings reveal a significant link between early allograft function risk scores and the percentage change in liver tissue ATG5 after D-HOPE, but not after NMP, confirming that excessive autophagy has a damaging effect on the liver [28,29]. This is because excessive autophagy, similar to an exacerbated inflammatory response, can damage cells by erroneously targeting normal organelles or proteins [28]. Consequently, the superior outcomes of NMP compared to D-HOPE may be attributed to NMP ability to replicate physiological conditions, support cellular metabolism, remove toxic metabolites, and provide real-time liver function monitoring, all of which enhance graft viability and contribute to improved results.

As expected, there was a significant build-up of pro-inflammatory cytokines, such as IL-6, IL-8, and TNF-α, in the perfusate during NMP, whereas no increase was observed during D-HOPE. NMP induces inflammation through metabolism reactivation, oxygen reintroduction and immune cell presence [30,31,32]. Recently, Ohman et al. [33] demonstrated that human livers with sufficient hepatocellular function during NMP show early activation of the innate immune response, immunogenic modification of donor livers before transplantation, and modulation of immune responses. This implies a potential influence of NMP on modifying the immunogenicity of donor livers before LT. Nevertheless, the occurrence of EAD was not correlated with inflammatory cytokine levels in the perfusate or percentage increases after perfusion.

Another expected result is the activation of regenerative mechanisms during NMP compared with D-HOPE. Liver regeneration is a highly orchestrated and complex process that involves a dynamic interplay between growth factors, cytokines, and cellular signaling pathways [34,35]. Following liver injury, hemodynamic changes and remodeling of the extracellular matrix are triggered, leading to the release of critical growth factors and the activation of specific molecular pathways [36]. HGF, released from the extracellular matrix in response to liver damage, rapidly increases due to the activation of damage-induced serine proteases. Its levels rise rapidly during NMP, where physiological temperatures activate the enzymatic activity of damage-induced serine proteases, but remain low during D-HOPE due to suppressed enzymatic activity at low temperatures.

Another key player in liver regeneration is OPN, a multifunctional glycoprotein with significant roles in inflammation, cell proliferation, and survival [37]. Notably, elevated OPN levels have been observed during normothermic machine perfusion (NMP). [37]. OPN supports liver regeneration by reducing apoptosis, promoting hepatocyte proliferation, and modulating immune responses [37]. This intricate coordination between hemodynamic changes, matrix remodeling, and growth factor activation underlines the complex nature of liver regeneration observed during NMP, whereas it is completely absent during HMP.

## 5. Conclusions

In summary, in comparing the distinct preservation and perfusion techniques of NMP and D-HOPE after NRP, our focus was on identifying the predictors of damage. This study emphasized the importance of carefully interpreting biomarkers and considering important differences in perfusion fluid composition to avoid prediction errors and associations with perfusion techniques. Our data support the use of FMN, an easy-to-measure and inexpensive biomarker, during D-HOPE, whereas ARG-1, although more expensive, could be used more universally during any type of MP, including NRP. Since the AUC for ARG-1 may lack sufficient robustness for independent clinical application, it emphasizes the importance of conducting further validation studies. The AUC observed for ARG-1 may not be robust enough for independent clinical application, highlighting the need for further validation studies. This study has limitations, notably the restricted sample size of DCD numbers compared with the two perfusion systems. The limited number of patients precluded the execution of multivariate analyses for predictive assessment, allowing only univariate analyses to be conducted. To assess graft viability and predict outcomes more accurately, further research is essential to identify reliable biomarkers that could supplement or replace those currently in use.

## Figures and Tables

**Table 1:** Baseline donor and recipient characteristics, and clinical outcomes.

	Transplanted (n = 32)	NMP (n = 16)	D-HOPE (n = 16)	*p*
Donor characteristics				
cDCD/uDCD, n	26/6	12/4	14/2	0.653
Male sex, n	24	13	11	0.414
Age, years	60.5 (49–70)	60.5 (48.5–67)	60.5 (49.5–70.5)	0.860
BMI, kg/cm^2^	26.1 ± 3.8	26.6 ± 4.5	25.5 ± 2.3	0.407
Last labs before NRP				
Glucose (mg/dL)	296 (260–360)	307 (263–361)	281 (224–342)	0.429
AST (IU/L)	51 (31.5–133)	48 (32–117)	55.5 (28.5–128)	0.456
ALT (IU/L)	51 (20.5–114)	36.5 (23–73.5)	66.5 (20.5–140)	0.963
Lactate (mmol/L)	12.4 ± 4.5	11.0 ± 2.5	13.9 ± 5.6	0.070
*Liver histology*				
Macrosteatosis %	5 (0–9)	5 (0–6.5)	5 (0.5–10)	0.628
Microsteatosis %	5 (4–10)	5 (3.5–10)	5 (4–10)	0.327
Necrosis %	0 (0–0)	0 (0–0)	0 (0–0)	0.878
Fibrosis stage 1–2, n	10	5	5	1.000
*NRP and procurement*				
No-flow time, min.	38 ± 16	37.7 ± 20	38 ± 4.2	0.987
cDCD WIT (f-WIT + no flow), min.	41 ± 10.2	40 ± 9.7	44 ± 11	0.656
uDCD WIT (low flow + no flow), min.	143 ± 7	147 ± 5.7	137 ± 3	0.084
NRP duration, min.	303.8 ± 52.9	318.1 ± 51.0	289.4 ± 52.5	0.127
*Ex situ machine perfusion*				
Liver weight pre-MP, grams	1517 ± 304	1485 ± 246	1549 ± 359	0.561
Liver weight post-MP, grams	1567 ± 305	1525 ± 246	1609 ± 358	0.446
CIT, min.	292 ± 55	294 ± 65	291 ± 46	0.872
Re-cooling time, min.	22 ± 10	25 ± 8.4	19 ± 10	0.098
MP duration, min.	243 ± 61	275 ± 56	210 ± 43	**0.001**
**Recipient characteristics**				
Male sex, n (%)	28 (87)	14 (87)	14 (87)	1.000
Age, years	62 (56–66)	58 (53.5–63.5)	64 (57.5–67.5)	0.060
BMI, kg/cm^2^	26.6 ± 3.3	26.4 ± 3.4	26.8 ± 3.3	0.707
MELD	11.7 ± 4.6	12.1 ± 5.1	11.4 ± 4.2	0.652
HCC, n	18	9	9	1.000
HCV, n	9	7	2	0.116
HBV, n	13	6	7	0.990
ASH, n	12	5	7	0.715
NASH, n	13	5	8	0.472
Caroli’s disease, n	1	0	1	0.990
Polycystic, n	1	1	0	0.990
*Postoperative outcomes*				
EAD, n	9	3	6	0.431
Ischemic cholangiopathy, n	0	0	0	1.000
EASE	−4.04 ± 1.43	−3.79 ± 1.82	−4.29 ± 0.891	0.338
L-GRAFT	−1.81 ± 1.1	−2.13 ± 0.62	−1.49 ± 1.38	0.238
Primary non-function, n	0	0	0	1.000
Six months of graft survival, n	29	16	13	0.225
Post reperfusion syndrome, n	12	6	6	1.000
Acute kidney injury, n	4	1	3	0.599
CCI at discharge	25.3 ± 26.6	23.9 ± 14.6	26.7 ± 35.9	0.779
ICU stay, days	9.5 ± 18.8	4.6 ± 2.6	14.7 ± 26.3	0.137
Hospital stay, days	18.6 ± 18.2	14.8 ± 6.1	22.5 ± 25.3	0.248
Vascular complications, n	0	0	0	1.000
Biliary complications–leakage, n	4	1	3	0.285

Data are expressed as mean ± standard deviation, median (interquartile range), and numbers. Abbreviations: ALT: alanine-amino-transferase; ASH: alcoholic steatohepatitis; AST: aspartate-amino-transferase; BMI: body mass index; CCI: Clavien comprehensive index; cDCD/uDCD: controlled donations after circulatory death/uncontrolled donations after circulatory death; CIT: cold ischemia time; D-HOPE: dual hypothermic machine perfusion; EAD: early allograft dysfunction; EASE: early allograft failure simplified estimation; f-WIT: functional warm ischemia time; HBV: hepatitis B virus; HCC: hepatocarcinoma; HCV: hepatitis C virus; ICU: intensive care unit; L-GRAFT: liver graft assessment following transplantation; MP: machine perfusion; MELD: model end-stage liver disease; NMP: normothermic machine perfusion; NRP: normothermic regional perfusion; NASH: non-alcoholic steatohepatitis; WIT: warm ischemia time. Bold indicates the *p* values < 0.05.

## Data Availability

The original contributions presented in this study are included in the article/Supplementary Materials. Further inquiries can be directed to the corresponding author.

## Supplementary Materials

The following supporting information can be downloaded at: https://www.mdpi.com/article/10.3390/biomedicines13010244/s1, Table S1: List of analytes analyzed in plasma and perfusion fluids, their range, sensitivity, unit of measure and Company; Figure S1: Dynamic change of (A) Lactate, (B) Glucose, (C) ALT and (D) AST during NRP, end-ischemic MPs and in recipients until a week post-transplant. Values are presented as mean ± SE. * Significant changes over time within each group (Friedman’s test with Bonferroni correction). Only values of *p* < 0.05, considered as significant, are reported; Figure S2: Comparison of ARG-1 values between D-HOPE and NMP in discarded grafts during end-ischemic MPs. Values are presented as mean ± SE. Only values of *p* < 0.05, considered as significant, are reported; Figure S3: (A) Dynamic change of Fumarate during NRP, end-ischemic MPs and in recipients until a week post-transplant. Values are presented as mean ± SE. * Significant changes over time within each group (Friedman’s test with with Bonferroni correction). Only values of *p* < 0.05, considered as significant, are reported. (B) FMN values during end-ischemic MP, represented in two separate scales for D-HOPE and NMP to underline the different and significant trends In addition, Supplementary Materials report details on donor selection, NRP management, ex-situ machine perfusion, histology, allocation and transplant, key definitions and outcome measures, RT-qPCR [38,39,40,41,42,43,44,45,46,47,48,49,50,51].

## Abbreviations

ALT: alanine-aminotransferase; ARG-1, arginase 1; AST, aspartate-aminotransferase; ATG-5, autophagy-related gene-5; DCD, donation after cardiocirculatory death; EAD, early allograft dysfunction; FMN, flavin mononucleotide; D-HOPE, dual hypothermic oxygenated machine perfusion; HGF, hepatocyte growth factor; IL-6/-8, interleukin-6/-8; IRI, ischemia-reperfusion injury; LT, Liver Transplantation; NADH, reduced nicotinamide adenine dinucleotide; NMP, normothermic machine perfusion; NRP, normothermic regional perfusion; OPN, osteopontin; TBARS, thiobarbituric acid reactive substances; TNF-α, tumor necrosis factor-α; -GST-α, glutathione-S-transferase- α; WIT, warm ischemia time.
