# Supplementary material for: Perfusate Liver Arginase 1 Levels After End-Ischemic Machine Perfusion Are Associated with Early Allograft Dysfunction
Source: Biomedicines. 2025 Jan 20;13(1):244. doi: 10.3390/biomedicines13010244 (PMC11760452; doi:10.3390/biomedicines13010244)
Supplement: Supplementary file 1 [file biomedicines-13-00244-s001.zip › biomedicines-3407724-supplementary.pdf]

Supplementary Materials

Perfusate Liver Arginase 1 Levels After End-Ischemic Machine Perfusion Are Associated with Early Allograft Dysfunction

1. Donor selection details

The study was conducted in a hospital setting at the Division of Liver Surgery and Transplantation and at a research facility of the Institute of Clinical Physiology of the National Research Council, both in Pisa, Italy, from December 2020 to September 2024. Data are stored within the IT systems of both Institutions, adhering to General Data Protection Regulation guidelines.

DCD donors were categorized based on the modified Maastricht classification proposed during the 2013 6th International Conference on Organ Donation after Circulatory Death [1]. The utilization of NRP during liver procurement adhered to the established Italian guidelines [2].

Donors called cDCD are individuals who require mechanical ventilation due to severe brain injury but do not meet the criteria for brain death. The decision to discontinue treatment for these patients is based on the perceived futility of further therapeutic interventions, irrespective of organ donation considerations. Heparin was administered at a dose of 300 IU/kg when the systolic blood pressure fell below 50 mm Hg.

Donors called uDCDs are potential donors who experience out-of-hospital cardiac arrest, undergo onsite cardiopulmonary resuscitation, and are transferred to the hospital with mechanical chest compression (LUCAS-Jolife AB/Physio-Control, Lund, Sweden). The peculiarity of uDCDs is that the ischemic time (as the interval between cardiac arrest and NRP start) is often extended, making the assessment of ischemic reperfusion injury difficult. In accordance with Italian law, death was officially pronounced after a flat EKG was observed for 20 minutes following the withdrawal of life-sustaining treatment. Time intervals were defined as follows: Time from the withdrawal of life-sustaining treatment to the initiation of NRP was referred to as T-WIT (Time Withdrawal of life-sustaining

treatment to Normothermic Regional Perfusion start), functional WIT (f-WIT) was defined as the time from when systolic blood pressure dropped below 50 mmHg or oxygen saturation fell below 70% to the initiation of NRP, and no-flow (NF) time represented the duration from cardiac arrest to the start of NRP [2-4]The no-flow time was the period from cardiac arrest to manual or mechanical chest compression in uDCD or from cardiac arrest to the start of NRP in cDCD, plus the time required for death declaration (20 minutes); in uDCD, the low-flow time was defined as the period of chest compression, whilst the total warm ischemia time (t-WIT) was the time from out-of-hospital cardiac arrest to start of NRP.

According to the Milan criteria [5], uDCDs and cDCDs were considered eligible for procurement if the following criteria were satisfied:

1. no absolute contraindication as per the Italian Transplant Agency
2. NRP flow > 2.0 L/minute
3. acceptable gross appearance at procurement
4. at least two of the following: 1) t-WIT ≤ 170 minutes for uDCD or f-WIT ≤ 120 minutes for cDCDs; 2) NRP peak alanine aminotransferase (ALT) <1000 IU/L; and 3) downward lactate trend during NRP.

All donors were procured using simultaneous aortic and portal flushes [6]. Donors received an extra 30.000 IU heparin before cross-clamping if the activated clotting time was >250 s. Although most organ donors are at risk of intravascular volume depletion because of the loss of vascular tone and increased capillary permeability, to limit potential bias related to excessive dilution during NRP or to NRP management, the donors included in the study did not receive more than two packets of red blood or one additional liter of colloidal fluids. Livers from both controlled (37 cDCD) and uncontrolled (20 uDCD), were included in the study. Briefly, cDCDs are patients with a particularly unfavourable medical prognosis, most frequently due to severe brain injury, in which cardiac death after the withdrawal of intensive care treatment, is anticipated. Instead, uDCDs are patients who experience out-of-hospital cardiac arrest, undergo onsite cardiopulmonary resuscitation, and are transferred to the hospital with mechanical chest compression (LUCAS-Jolife AB/Physio-Control, Lund, Sweden) [3]. These patients are considered potential donors once resuscitation proves unsuccessful or irreversible brain damage is diagnosed.

2. NRP Management

In Italy, donors with DCD do not undergo fast recovery, and the use of NRP is obligatory in all cases. NRP is initiated by percutaneous cannulation of the femoral vessels on one side, with premortem insertion of Seldinger wires permitted, but not full-vessel cannulation. An occlusion balloon was placed through the contralateral femoral artery and inflated at the level of the supra-celiac aorta to prevent perfusion of the heart and the brain. Correct positioning of the balloon is confirmed through chest X-rays and/or trans-oesophageal echocardiography.

The NRP circuit is primed with 800–1000 mL of Ringer's acetate solution, and pump flow rates are maintained above 2 L/min/m^2. The arterial oxygen tension (PO_2) is maintained at 80–90 mm Hg, arterial carbon dioxide tension (PCO_2) at 35–40 mm Hg, and the perfusion temperature is maintained at 37°C. NRP is sustained using a membrane oxygenator, a heat exchanger, and a cardiopulmonary bypass pump, which can be either the Rotaflow Console or the Cardiohelp System (Maquet, Rastatt, Germany). During NRP, transaminases, a full arterial gas profile, and lactate levels are regularly monitored, typically every hour [5, 7].

Liver biopsy during procurement is mandatory, and grafts are discarded if any of the following conditions are present: macro-vesicular steatosis exceeding 30%, fibrosis exceeding a score of 2 according to Ishak's classification, or necrosis exceeding 10%. Micro-steatosis is evaluated but not used in the assessment of graft viability [5, 7].

After NRP, the livers are flushed with a cold preservation solution through both the aorta and portal vein [2-4]. Subsequently, they are transported to the transplant center for back-table preparation and ex-situ MP.

3. Ex-situ machine perfusion

Once considered eligible for organ transplant, DCD grafts were procured and randomized to D-HOPE or NMP in a 1:1 ratio using the Sealed Envelope system. The random assignment sequence was generated by an independent statistician using the Sealed Envelope web

platform. Grafts were enrolled in the study by the surgical team responsible for procurement.

3.1 D-HOPE management

The perfusion system was initially prepared with 4 liters of Belzer University of Wisconsin machine perfusion solution (UW-MPS) (Bridge for Life, Ltd. in Columbia, SC). Arterial and portal pressures were regulated to 25 mm Hg, employing a pulsatile flow for the former and a continuous flow for the latter, maintaining pressures at 3-4 mmHg. An oxygen flow rate of 0.25 liters per minute was established. The desired temperature range for the liver was set between 4°C and 10°C. The temperature of the graft was continuously monitored using a custom-made probe placed on the liver's surface, while the temperature of the perfusate was tracked through built-in probes. DHOPE was sustained until the recipient's hepatectomy was completed. Notably, during the hypothermic perfusion phase, no administration of electrolytes, glucose, or medications was deemed necessary.

3.2 NMP management

The management of NMP at our center follows a protocol previously detailed [8, 9]. Briefly, the grafts are subjected to perfusion at 37°C while under medical supervision, using a blood-based perfusate. The initial temperature of the perfusate is set to 25°C and is gradually increased by 1°C every 2 minutes. Oxygenation is facilitated through an anesthesia ventilator, initially configured to deliver 2 liters per minute with a 30% fraction of inspired oxygen. Subsequent adjustments are made based on the pH, oxygen, and carbon dioxide partial pressures within the perfusate. Blood gas analyses are conducted at 20-minute intervals during the initial hour and subsequently at 30-minute intervals. The objective is to maintain physiological pH and ion levels, along with a partial pressure of oxygen ranging from 100 to 150 mmHg.

Throughout NMP, measurements are taken for perfusate glucose, transaminases, and lactate, as well as the production and quality of bile (pH, sodium levels, glycemia, lactate, and $HCO3^-$).

Supplemental glucose (10 ml at 33%) is introduced when the perfusate level falls below 150 mg/ml. After the recipient hepatectomy is completed, the grafts are flushed with 2 L of cold Servator (S.A.L.F. S.p.A. Laboratorio Farmacologico, Bergamo, Italy) through both the hepatic artery and portal vein, allowing for re-cooling.

Liver graft weight is documented both before and after Machine Perfusion (MP). Grafts are deemed suitable for transplantation if they meet the following criteria: 1) demonstrated a reduction in lactate levels within the perfusate, regardless of the initial and final values, 2) acceptable gross appearance characterized by uniform vascularization, and 3) stable flows. Bile production and quality are evaluated but not considered for viability assessment, unless the 1-hour ALT perfusate level exceeds 5000 IU/L.

4. Histology

Liver biopsies were obtained at four stages: procurement, post-back-table, after-machine perfusion (MP), and post-liver transplantation (LT) [3, 4]. These samples were thinly sectioned and stained with hematoxylin and eosin (H&E). Key features assessed included sinusoidal congestion, vacuolization of hepatocyte cytoplasm, and parenchymal necrosis.

5. Allocation and transplant

Recipients aged 18 or older were deemed eligible for a DCD graft. Adherence to Italian national guidelines [10] guided patient evaluations, and informed consent was mandatory for accepting a DCD liver, both at the time of wait-listing and upon the availability of a potential DCD graft.

DCD livers meeting NRP and ex-situ viability criteria were prioritized for allocation to low-risk recipients with hepatocellular carcinoma (HCC) and shared regionally under the Italian allocation scheme. Extremely urgent patients and those with a model for end-stage liver disease (MELD) score ≥30 points were excluded. Liver transplants were conducted using either conventional or piggy-back techniques, with or without veno-venous bypass, depending on the surgeon's preference. Duct-to-duct biliary anastomosis was routinely performed with a T-tube, and removed three months post-transplantation.

Immunosuppression for non-HCV-RNA positive patients was based on anti-CD25 induction, tacrolimus, mycophenolate mofetil, and steroids [4].

6. Key Definitions and Outcome Measures

Cold ischemia time was defined as the time from cold flush to MP start. The model for-end-stage liver disease (MELD) score was calculated as laboratory MELD without the inclusion of exception points for HCC. Early allograft dysfunction (EAD) as per Olthoff et al. [11] Postreperfusion syndrome and acute kidney injury were defined according to Aggarwal et al. [12] and KDIGO guidelines respectively [13]. Postoperative complications were classified according to Clavien-Dindo [14] and scored using the comprehensive complication index [15], early allograft failure simplified estimation (EASE) and liver graft assessment following transplantation (L-GRAFT) risk scores were calculated as reported [16].

7. Tissue protein assays and quantitative Reverse Transcription Polymerase Chain Reaction (RT-qPCR)

Tissue specimens were cut into two parts: one part was solubilized in lysis buffer for protein assays in a 50 mM Tris-HCl buffer at pH 8 containing 0.5% Triton, and 0.25% Deoxycholic Acid. The other piece was disrupted in QIAzol® Lysis Reagent (Qiagen, S.p.A, Milano, Italy) for RNA extraction. Both tissue pieces were homogenized with Tissue Lyser II (Qiagen) to disrupt biological samples through high-speed shaking in plastic tubes with stainless steel beads for 2 minutes.

The resulting protein extracts were then centrifuge for 10 minutes at 16,000g and the detergent was removed by columns (Pierce, Thermo Scientific, USA). A total protein assay was carried out as previously described [3].

Total RNA was extracted using miRNeasy Mini Kit (Qiagen), according to manufacturer's instructions. RNA concentrations and purity were determined spectrophotometrically using NanoDrop (ThermoFisher Scientific). The reverse transcription of mRNA to cDNA was performed using IScript cDNA Synthesis Kit (Bio-Rad, Hercules, California, USA) optimized for reliable cDNA synthesis over 1 μg of mRNA as a template.

RT-qPCR was performed in a Rotor-GeneQ (Qiagen) using gene-specific oligonucleotide primers for custom probes (ThermoFisher Scientific). The reaction mixture for each sample contained 10 μL of Master Mix (QuantiNova SYBR Green PCR Kit - Qiagen), 1 μL of forward primer, 1 μL of reverse primer and 1 μL of cDNA sample. The final volume of the reaction mixture was adjusted to 20 μL with RNAse-free water.

The real-time PCR thermal cycling conditions in the Rotor-GeneQ for ATG-5 were as follows: initial denaturation at 95°C for 10 min, followed by 40 amplification cycles at 95°C for 15 seconds and then 60°C for 60 seconds. Instead, the thermal cycling conditions for ACTB were: initial denaturation at 95°C for 15 min, followed by 40 amplification cycles at 94°C for 10 seconds, 58°C for 10 seconds and then 72°C for 60 seconds. The forward primer sequence used for ATG-5 and ACTB were: 5′- GCTTCGAGATGTGTGGTTTGG- 3′ and 5′- CATGTACGTTGCTATCCAGGC - 3′.

Gene expression was normalized with ACTB. Individual relative gene expression values were calculated using the following formula: $2^{-(\text{Ct gene of interest} - \text{Ct constitutive gene})}$.

8. Supplemental Table

Table S1. List of analytes analyzed in plasma and perfusion fluids, their range, sensitivity, unit of measure and Company.

**Analyte**	**Assay range**	**Sensitivity**	**Units**	**Company**
α-GlutathioneS-Transferase/α-GST*	14–10000	4.0	pg/mL	EMD Millipore Corporation, Billerica, MA
Liver-type Arginase 1/ARG-1*	69–50000	70.7	pg/mL	ʺ
Interleukin-6/IL-6*	0.68–500	0.2	pg/mL	ʺ
Interleukin-8/IL-8*	1.3–1000	0.3	pg/mL	ʺ
Hepatocyte Growth Factor/HGF*	27.4–20000	6.8	pg/mL	ʺ
Osteopontin/OPN*	548.6–400000	285.3	pg/mL	ʺ
Fumarate°	23–116	-	ng/μL	Sigma-Aldrich, St. Louis, MO
Succinate°	80–400	-	ng/μL	ʺ

* Method: Human Magnetic Luminex® Screening Assay

° Method: Colorimetric Assay

9. Supplementary Figures

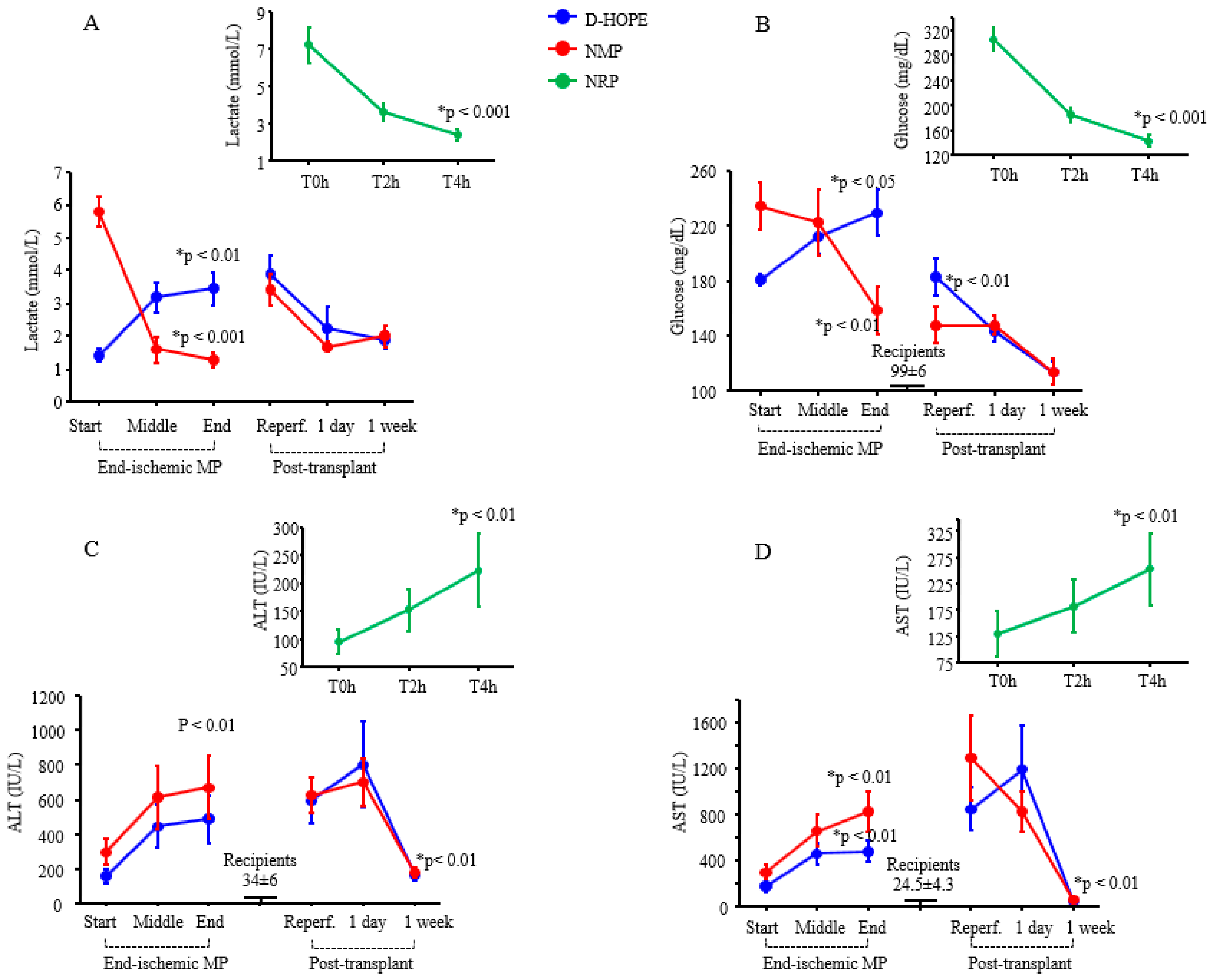

Figure S1. Dynamic change of (A) Lactate, (B) Glucose, (C) ALT and (D) AST during NRP, end-ischemic MPs and in recipients until a week post-transplant. Values are presented as mean ± SE. *Significant changes over time within each group (Friedman's test with Bonferroni correction). Only values of P <0.05, considered as significant, are reported.

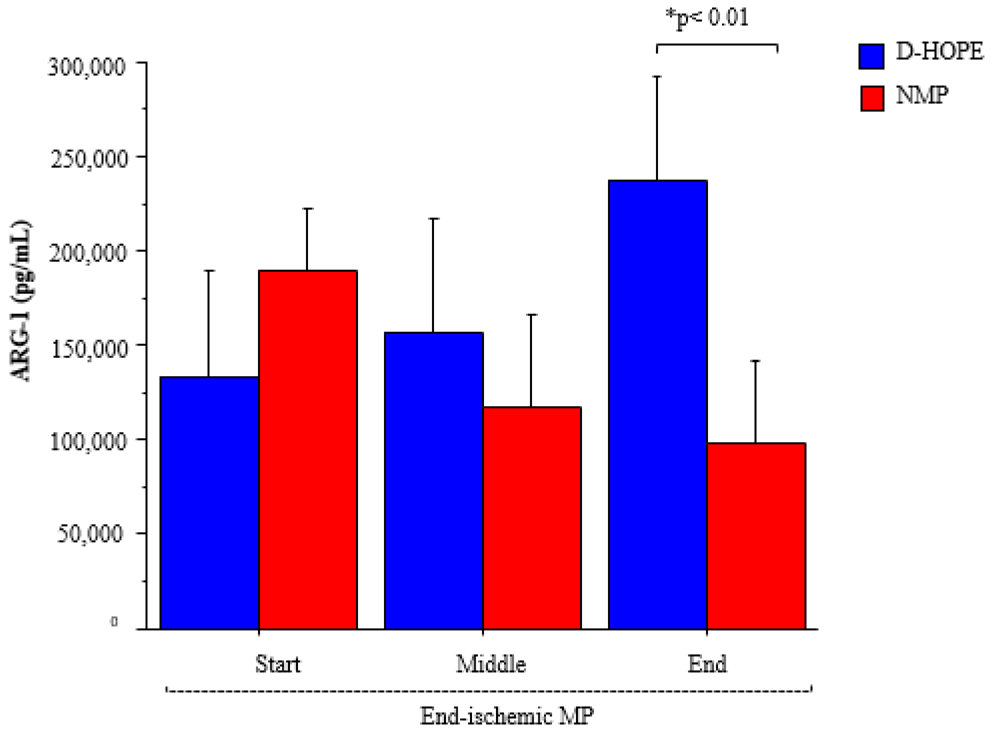

Figure S2. Comparison of ARG-1 values between D-HOPE and NMP in discarded grafts during end-ischemic MPs. Values are presented as mean ± SE. Only values of P <0.05, considered as significant, are reported.

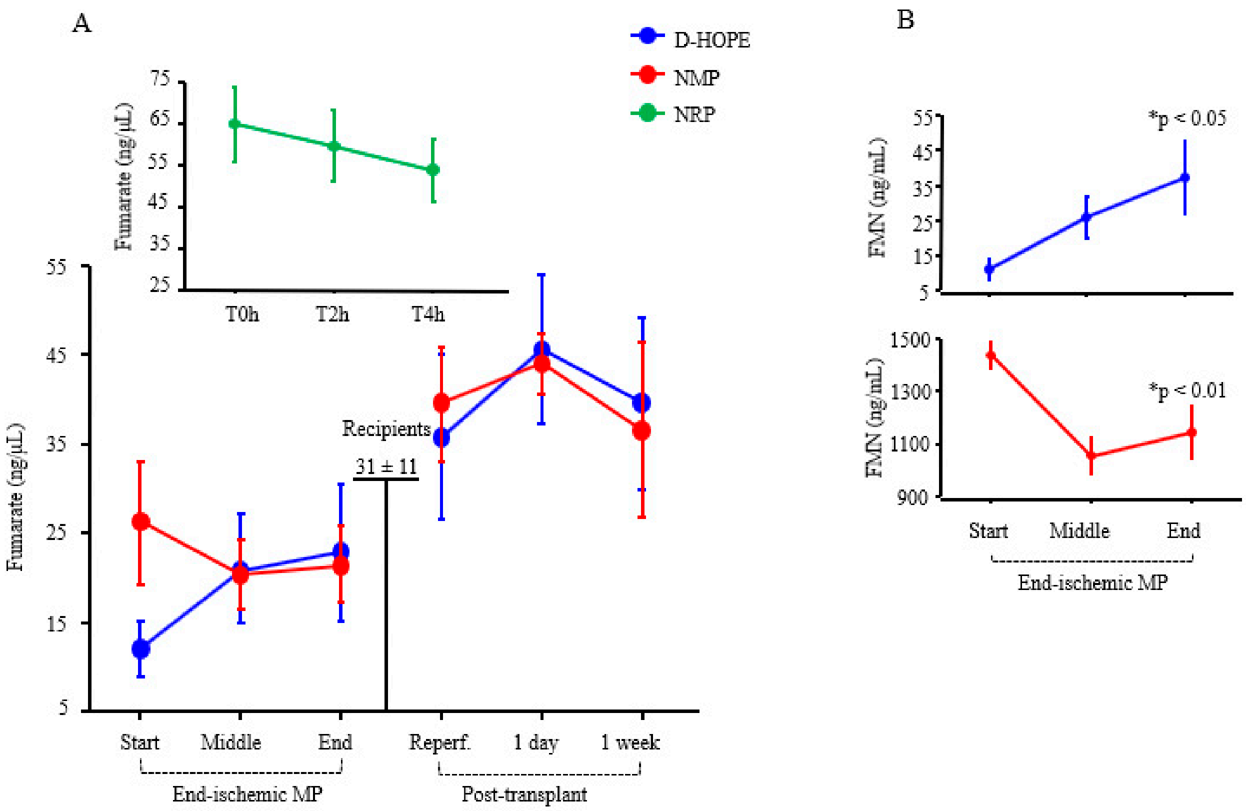

Figure S3. (A) Dynamic change of Fumarate during NRP, end-ischemic MPs and in recipients until a week post-transplant. Values are presented as mean ± SE. *Significant changes over time within each group (Friedman's test with Bonferroni correction). Only values of P <0.05, considered as significant, are reported. (B) FMN values during end-ischemic MP, represented in two separate scales for D-HOPE and NMP to underline the different and significant trends.

References

1. Thuong, M.; Ruiz, A.; Evrard, P.; Kuiper, M.; Boffa, C.; Akhtar, M. Z.; Neuberger, J.; Ploeg, R. New classification of donation after circulatory death donors definitions and terminology. *Transpl Int* **2016**, *29*, 749-759.
2. Ghinolfi, D.; Lai, Q.; Dondossola, D.; De Carlis, R.; Zanierato, M.; Patrono, D.; Baroni, S.; Bassi, D.; Ferla, F.; Lauterio, A.; et al. Machine Perfusions in Liver Transplantation: The Evidence-Based Position Paper of the Italian Society of Organ and Tissue Transplantation. *Liver Transpl* **2020**, *26*, 1298-1315.
3. Basta, G.; Melandro, F.; Babboni, S.; Del Turco, S.; Ndreu, R.; Torri, F.; Martinelli, C.; Silvestrini, B.; Peris, A.; Lazzeri, C.; et al. An extensive evaluation of hepatic markers of damage and regeneration in controlled and uncontrolled donation after circulatory death. *Liver Transplantation* **2023**, *29*, 813-826.
4. Torri, F.; Balzano, E.; Melandro, F.; Maremmani, P.; Bertini, P.; Lo Pane, P.; Masini, M.; Rotondo, M. I.; Babboni, S.; Del Turco, S.; et al. Sequential Normothermic Regional Perfusion and End-ischemic Ex Situ Machine Perfusion Allow the Safe Use of Very Old DCD Donors in Liver Transplantation. *Transplantation* **2024**, *108*, 1394-1402.
5. De Carlis, R.; Schlegel, A.; Frassoni, S.; Olivieri, T.; Ravaioli, M.; Camagni, S.; Patrono, D.; Bassi, D.; Pagano, D.; Di Sandro, S.; et al. How to Preserve Liver Grafts From Circulatory Death With Long Warm Ischemia? A Retrospective Italian Cohort Study With Normothermic Regional Perfusion and Hypothermic Oxygenated Perfusion. *Transplantation* **2021**, *105*, 2385-2396.
6. Ghinolfi, D.; Tincani, G.; Rreka, E.; Roffi, N.; Coletti, L.; Balzano, E.; Catalano, G.; Meli, S.; Carrai, P.; Petruccelli, S.; et al. Dual aortic and portal perfusion at procurement prevents ischaemic-type biliary lesions in liver transplantation when using octogenarian donors: a retrospective cohort study. *Transplant International* **2019**, *32*, 193-205.
7. Ghinolfi, D.; Dondossola, D.; Rreka, E.; Lonati, C.; Pezzati, D.; Cacciatoinsilla, A.; Kersik, A.; Lazzeri, C.; Zanella, A.; Peris, A.; et al. Sequential Use of Normothermic Regional and Ex Situ Machine Perfusion in Donation After Circulatory Death Liver Transplant. *Liver Transpl* **2021**, *27*, 385-402.
8. Ghinolfi, D.; Rreka, E.; De Tata, V.; Franzini, M.; Pezzati, D.; Fierabracci, V.; Masini, M.; Cacciatoinsilla, A.; Bindi, M. L.; Marselli, L.; et al. Pilot, Open, Randomized, Prospective Trial for Normothermic Machine Perfusion Evaluation in Liver Transplantation From Older Donors. *Liver Transpl* **2019**, *25*, 436-449.
9. Pezzati, D.; Ghinolfi, D.; Balzano, E.; De Simone, P.; Coletti, L.; Roffi, N.; Rreka, E.; Meacci, L.; Campani, D.; Mazzoni, A.; et al. Salvage of an Octogenarian Liver Graft Using Normothermic Perfusion: A Case Report. *Transplant Proc* **2017**, *49*, 726-728.
10. Costa, A. N.; Grossi, P.; Castiglione, A. G.; Grigioni, W. F. Quality and safety in the Italian donor evaluation process. *Transplantation* **2008**, *85*, S52-S56.
11. Olthoff, K. M.; Kulik, L.; Samstein, B.; Kaminski, M.; Abecassis, M.; Emond, J.; Shaked, A.; Christie, J. D. Validation of a current definition of early allograft dysfunction in liver transplant recipients and analysis of risk factors. *Liver Transpl* **2010**, *16*, 943-949.
12. Aggarwal, S.; Kang, Y.; Freeman, J. A.; Fortunato, F. L.; Pinsky, M. R. Postreperfusion syndrome: cardiovascular collapse following hepatic reperfusion during liver transplantation. *Transplant Proc* **1987**, *19*, 54-55.
13. Thomas, M. E.; Blaine, C.; Dawnay, A.; Devonald, M. A.; Ftouh, S.; Laing, C.; Latchem, S.; Lewington, A.; Milford, D. V.; Ostermann, M. The definition of acute kidney injury and its use in practice. *Kidney Int* **2015**, *87*, 62-73.
14. Dindo, D.; Demartines, N.; Clavien, P. A. Classification of surgical complications: a new proposal with evaluation in a cohort of 6336 patients and results of a survey. *Ann Surg* **2004**, *240*, 205-213.
15. Huang, H.; Zhang, Z.; Hao, H.; Wang, H.; Shang, M.; Xi, Z. The comprehensive complication index is more sensitive than the Clavien-Dindo classification for grading complications in elderly patients after radical cystectomy and pelvic lymph node dissection: Implementing the European Association of Urology guideline. *Front Oncol* **2022**, *12*, 1002110.
16. Chen, S. R.; Wang, T. L.; Luo, T.; He, S. J.; Huang, C. J.; Jia, Z. H.; Zhan, L. Q.; Wang, D. P.; Zhu, X. F.; Guo, Z. Y.; He, X. S. Prediction of Graft Survival Post-liver Transplantation by L-GrAFT Risk Score Model, EASE Score, MEAF Scoring, and EAD. *Front Surg* **2021**, *8*.
